# Stimulating prefrontal cortex facilitates training transfer by increasing representational overlap

**DOI:** 10.1093/cercor/bhae209

**Published:** 2024-05-20

**Authors:** Yohan Wards, Shane E Ehrhardt, Kelly G Garner, Jason B Mattingley, Hannah L Filmer, Paul E Dux

**Affiliations:** School of Psychology, The University of Queensland, McElwain Building, Campbell Road, St Lucia, Queensland 4072, Australia; School of Psychology, The University of Queensland, McElwain Building, Campbell Road, St Lucia, Queensland 4072, Australia; School of Psychology, The University of Queensland, McElwain Building, Campbell Road, St Lucia, Queensland 4072, Australia; Queensland Brain Institute, The University of Queensland, Building 79, Upland Road, St Lucia, Queensland 4072, Australia; School of Psychology, University of New South Wales, Mathews Building, Gate 11, Botany Street, Randwick, New South Wales 2052, Australia; School of Psychology, University of Birmingham, Hills Building, Edgbaston Park Rd, Birmingham B15 2TT, United Kingdom; School of Psychology, The University of Queensland, McElwain Building, Campbell Road, St Lucia, Queensland 4072, Australia; Queensland Brain Institute, The University of Queensland, Building 79, Upland Road, St Lucia, Queensland 4072, Australia; School of Psychology, University of Birmingham, Hills Building, Edgbaston Park Rd, Birmingham B15 2TT, United Kingdom; School of Psychology, The University of Queensland, McElwain Building, Campbell Road, St Lucia, Queensland 4072, Australia; School of Psychology, The University of Queensland, McElwain Building, Campbell Road, St Lucia, Queensland 4072, Australia

**Keywords:** tDCS, transfer, learning, machine learning, cognitive training

## Abstract

A recent hypothesis characterizes difficulties in multitasking as being the price humans pay for our ability to generalize learning across tasks. The mitigation of these costs through training has been associated with reduced overlap of constituent task representations within frontal, parietal, and subcortical regions. Transcranial direct current stimulation, which can modulate functional brain activity, has shown promise in generalizing performance gains when combined with multitasking training. However, the relationship between combined transcranial direct current stimulation and training protocols with task-associated representational overlap in the brain remains unexplored. Here, we paired prefrontal cortex transcranial direct current stimulation with multitasking training in 178 individuals and collected functional magnetic resonance imaging data pre- and post-training. We found that 1 mA transcranial direct current stimulation applied to the prefrontal cortex paired with multitasking training enhanced training transfer to spatial attention, as assessed via a visual search task. Using machine learning to assess the overlap of neural activity related to the training task in task-relevant brain regions, we found that visual search gains were predicted by changes in classification accuracy in frontal, parietal, and cerebellar regions for participants that received left prefrontal cortex stimulation. These findings demonstrate that prefrontal cortex transcranial direct current stimulation may interact with training-related changes to task representations, facilitating the generalization of learning.

## Introduction

Humans routinely demonstrate the ability to rapidly adapt to changes in our environment. Such behavior requires learning generalization—the transfer of skills from 1 task to another—which has been the subject of psychological research for over a century (Woodworth and Thorndike 1901). Despite our competency in flexibly applying our cognition to a wide variety of tasks in everyday life, we demonstrate substantial performance decrements whenever we perform multiple tasks simultaneously, even when they are simple (Telford 1931; Pashler 1994; Marois and Ivanoff 2005). A fundamental trade-off between multitasking and learning generalization has recently been proposed whereby multitasking costs reflect impairments due to information sharing across tasks (Musslick and Cohen 2021; Garner and Dux 2023). Specifically, the brain exploits shared variability between tasks to learn new tasks more rapidly (Musslick and Cohen 2021). This allows faster generalization, but comes at the expense of multitasking ability, as both tasks require access to the same underlying task representation(s). To wit, by dynamically encoding and integrating sensory and motor information through abstract task rules in multiple-demand network regions (Garner et al. 2016; Vaidya et al. 2021), the brain can rapidly adapt to learning new tasks. However, this same processing architecture gives rise to multitasking limitations due to interference between overlapping task representations (Musslick et al. 2017). This trade-off creates an interesting dilemma as improved capacity for both multitasking and generalizable learning abilities are increasingly desirable in the modern world (Strobach 2020).

Training interventions are thought to mitigate multitasking costs through shifting the balance in this fundamental trade-off in the brain’s information processing capabilities. By reducing representational overlap within frontoparietal–subcortical (FP–SC) brain regions that subserve the constituent tasks (Dux et al. 2009; Watanabe and Funahashi 2014; Garner and Dux 2015; Marti et al. 2015; Strobach and Torsten 2017; Garner et al. 2020), training minimizes the competition for shared resources, resulting in enhanced performance on the trained task. Both trained task improvements and generalizability of these gains to untrained tasks are core aims of many brain training interventions in commercial settings (Green and Bavelier 2008; Owen et al. 2010), but evidence for the transfer of purely training-induced improvements to untrained tasks is mixed (Owen et al. 2010; Liepelt et al. 2011; Strobach et al. 2012; Garner et al. 2014; Sala and Gobet 2019). An approach that has shown promise in enhancing transfer involves combining non-invasive brain stimulation techniques, such as transcranial direct current stimulation (tDCS) with behavioral training interventions (Martin et al. 2013; Filmer, Lyons, et al. 2017; Ehrhardt et al. 2021).

Transcranial direct current stimulation, which generates an exogenous electric field that alters the activity of underlying neurons (Terzuolo and Bullock 1956; Stagg and Nitsche 2011) and glial cells (Monai et al. 2016), has shown promise in enhancing performance on untrained tasks when paired with training on working memory (Martin et al. 2013; Trumbo et al. 2016; Ruf et al. 2017), cognitive flexibility (Brem et al. 2018), mathematics (Looi et al. 2016), and multitasking (Filmer, Lyons, et al. 2017; Ehrhardt et al. 2021). In a related study, Filmer, Varghese, et al. (2017) found that anodal stimulation applied to the left prefrontal cortex (PFC) during learning resulted in faster evidence accumulation for both the training task and visual search transfer task. This suggests that the addition of tDCS to training may improve information processing efficiency on more than just the trained task. Given the recent theoretical and computational advances in understanding the interplay between the mechanisms underlying multitasking costs and learning generalization (Bartolo et al. 2020; Musslick and Cohen 2021; Garner and Dux 2023), combining left PFC tDCS with multitasking training provides a unique avenue for exploring the neural substrates of transfer.

Here, using multivariate pattern analysis, we sought to investigate how changes in informational overlap for the trained task may underlie tDCS induced transfer to a spatial attention task. Specifically, we assessed whether the performance transfer elicited by combined training and tDCS can be attributed to greater overlap in task representations, facilitating the sharing of information across tasks. To this end, we used ultra-high field (7T) functional magnetic resonance imaging (fMRI) to examine how patterns of functional brain activity are linked with the effects of combined multitasking training and tDCS. Capitalizing on the individual variability commonly observed in both the behavioral response to tDCS (López-Alonso et al. 2014; Filmer et al. 2019) and the neural response to tasks (Osaka et al. 2003; Crittenden and Duncan 2014), we examined the extent to which changes in task representations associated with our interventions underpin performance transfer. Our findings revealed that PFC tDCS combined with multitasking training induced transfer to an untrained visual search task. Among participants who received left PFC tDCS, those who showed a decrease in decoding accuracy in superior parietal, orbitofrontal, and cerebellar regions after multitasking training demonstrated greater performance on the visual search task. These findings suggest that the overlapping nature of task representations during multitasking may mediate performance transfer, and therefore may reflect a neural substrate of generalizable learning.

## Materials and methods

### Study design

**Fig. 1:**
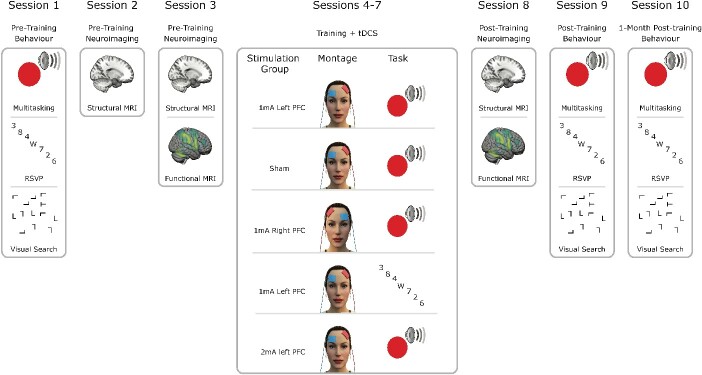
Summary of experimental protocol. Pre-training behavioral and neuroimaging sessions were followed by 4 d of combined tDCS and cognitive training according to group allocation. Four groups trained on multitasking and received either sham stimulation, 1 mA left hemisphere PFC stimulation, 1 mA right PFC stimulation, or 2 mA left PFC stimulation. Another group trained on a control training task, RSVP while receiving 1 mA left PFC stimulation. Immediately following these combined stimulation and training sessions were post-training neuroimaging and behavioral sessions. Finally, 1 month later, there was another behavioral assessment. For the full protocol including other scans and behavioral assessments beyond the scope of the current study, see Wards et al. (2023).

The study design was preregistered at the Open Science Framework (https://tinyurl.com/5h8u72j5). The study comprised 10 sessions: pre-training, post-training, and follow-up behavioral testing sessions; 4 sessions combining tDCS with training; and 3 imaging sessions (see Fig. 1). The imaging sessions involved initial magnetic resonance spectroscopy, T1-weighted (T1w), fluid attenuated inversion recovery, quantitative susceptibility mapping, and diffusion weighted structural scans. We also conducted task-based and resting-state functional scans before and after training. We focused on the task-based fMRI and T1w structural scans in the current study.

The testing sessions (pre-, post-, and follow-up) included an extensive set of 9 cognitive tasks aiming to test different cognitive processes, described in full in Wards et al. (2023). We conducted these testing sessions on 2014 model Mac mini computers (2.8 GHz Intel Core i5, OSX High Sierra v 10.13.6), with 24-inch ASUS VG248 monitors (144 Hz refresh rate) displaying the tasks. We implemented the tasks using MATLAB 2016b (The MathWorks, Inc., Matick, MA, USA) with custom code and the Psychtoolbox (Brainard 1997; Pelli 1997; Kleiner et al. 2007; see http://psychtoolbox.org). During the behavioral sessions, participants were positioned at ~57 cm from the screen.

We used a mixed design, dividing participants evenly among 5 groups while ensuring their participation in all sessions. Two groups—those receiving sham left PFC stimulation and those receiving 1 mA left PFC stimulation with single-/multi-task training—were double-blinded throughout the experiment. Note that all “targets” for stimulation refer to the anode electrode location. The remaining 3 groups acted as active controls: A group receiving 2 mA left PFC stimulation with single/multitask training served as a dosage control; a group receiving 1 mA stimulation to the right PFC with single/multitask training served as a montage control; and a group receiving 1 mA to the left PFC with rapid serial visual presentation (RSVP) task training served as a task control (active control group). To minimize the impact of task order on baseline performance measurements, we pseudorandomized and counterbalanced task order across all participants and groups.

### Participants

Our sample size and recruitment stopping rule were calculated using a power analysis via G*Power. This analysis was based on conservative effect sizes (Cohen’s *D* of 0.3) for the behavioral impacts of tDCS on multitasking training, as indicated by our laboratory’s prior studies (Filmer, Varghese, et al. 2017; Ehrhardt et al. 2021). This analysis revealed that 33 participants per group would provide 95% power for between-group comparisons, while 46 participants per group would be necessary for the same power for individual differences analyses. Therefore, we set a maximum goal of 250 participants (50 per group) to enable both between- and within-subjects analyses.

The coronavirus disease 2019 (COVID-19) pandemic and associated lockdowns resulted in a final sample size of 207 participants. Despite setting a stopping date of 2021 September 28, we continued data collection until 2022 January 15, to reach the minimum sample size for between-participants comparisons. This extension was decided without examining the data and was done based on the a priori power analysis. After excluding 29 subjects for reasons described below, we arrived at a final sample of 178 participants who completed sessions 1 through 9. Of these, 167 participants also completed session 10 (the 1-month follow-up).

### Participant exclusion and group allocation

We excluded 6 participants due to incidental findings (all pineal gland cysts) in their initial MRI, 10 participants who could not tolerate the MRI scans, and 3 participants who could not complete sessions due to COVID-related lockdowns. Other exclusions included 3 participants failing to meet the 60% accuracy threshold in the multitasking paradigms at baseline, 2 participants who missed sessions, and a few others due to personal reasons (1), inappropriate behavior (1), tDCS discomfort (1, no adverse effects noted), psychoactive medication use before a tDCS session (1), or significantly reduced accuracy in a training session (1, <30% accuracy during pre-training familiarization). Eight participants could not attend the 1-mo follow-up session due to COVID-19 lockdowns, and 3 did not return for testing.

Participants passed a tDCS safety screening questionnaire and a quiz to confirm their understanding of the study’s techniques (i.e. tDCS and MRI) before testing sessions. They also completed an MRI safety questionnaire before MRI sessions. We excluded potential participants before testing based on factors like history of brain trauma, current use of psychoactive medications, and personal or family history of epilepsy. The study was approved by The University of Queensland Human Research Ethics Committee, and all participants provided written informed consent for each session. Participants received compensation at a rate of $20 AUD/h, yielding ~$380 in total if all 10 sessions were completed. Participants were compensated for their time, regardless of study completion.

After session 1, participants were assigned to groups using a custom automated group assignment algorithm. This algorithm aimed to minimize potential baseline group differences by pseudo-randomizing participant allocation to groups, based on key characteristics such as age, sex, intervention time (AM or PM), single- and multitask reaction times (RTs), and task completion order.

### Stimulation protocol

Stimulation was delivered using a NeuroConn stimulator, which used 2 5 × 5 cm saline-soaked sponges with rubber electrodes secured by rubber straps. For individuals receiving left PFC stimulation, the anodal electrode was positioned 1 cm posterior to F3, and the cathodal electrode was placed over the right supraorbitofrontal cortex, in alignment with prior studies (Dedoncker et al. 2016; Filmer, Lyons, et al. 2017; Ehrhardt et al. 2021). Individuals in the right PFC stimulation group had the anodal electrode placed 1 cm posterior to F4, and the cathodal electrode over the left supraorbitofrontal cortex, mirroring the left hemisphere montage. Participants received 13 min of online stimulation during training, with a 30-s linear ramp-up and ramp-down. Given previous evidence suggesting brain stimulation outcomes can be influenced by time of day (Sale et al. 2007), participants completed their sessions at roughly the same time each day (±2 h). Our chosen intensities, 1 and 2 mA, are used in most tDCS studies (95%, Bikson et al. 2016), therefore facilitating generalization of dosage and montage effects to other findings. Furthermore, our laboratory has previously identified distinct effects of stimulation intensity on multitasking performance and decision-making processes (Ehrhardt et al. 2021, 2022).

### Behavioral data analysis

We used Bayesian methods to evaluate the strength of evidence in support or against null and alternative hypotheses concerning task performance in the cognitive battery before and after training. As stated in the pre-registration, we did not apply corrections for multiple comparisons, since the Bayesian approach provides more conservative comparisons than null hypothesis testing procedures, reducing the risk of type 1 errors (Gelman and Tuerlinckx 2000). We anticipated that some tasks and groups would show no training effect across our control groups and tasks; hence, we intended to evaluate the strength of evidence for these null effects. Bayes factors (BFs) for the alternative hypothesis (BF_10_) exceeding 3.2 indicated meaningful evidence in favor of the alternative hypothesis relative to the null, while a BF_10_ < 0.3125 signified meaningful evidence supporting the null hypothesis over the alternative (Kass and Raferty 1995).

The following procedures were established before conducting our key hypothesis tests. To ensure sufficient trial numbers for analysis and to confirm proper task comprehension and completion, we excluded participants from specific task analyses (Trained Multitask, Transfer Multitask, Dynamic Dual Task, Go No-Go, and Visual Search—see Ehrhardt et al. 2024) if their average accuracy for either the pre- or the post-training session fell below 60%. This is in line with our previous study protocols.

Subsequently, at the individual participant level, trials underwent an outlier removal analysis by calculating the interquartile range (IQR) for RT distributions, multiplying this range by 1.5, and adding this value to the third quartile (Q3 + 1.5 × IQR). RTs that exceeded this threshold were excluded for each participant. RTs faster than 0.2 s were also excluded, as per common practice.

We also applied the same Q3 + 1.5 × IQR exclusion threshold at the participant level for each task, where a mean RT exceeding this threshold for either the pre- or the post-training sessions resulted in that participant being excluded from analysis on the respective task. Our preregistration stated that we would use an exclusion threshold >3 standard deviations for the trained multitask and did not specify an approach for all other tasks. For the sake of consistency with the trial-level approach, we reported data using the IQR outlier analysis for all tasks. To assess the effect of combined training and tDCS on the trained multitasking paradigm, we employed Bayesian *t*-tests on the performance change (single- and multi-task RT, as well as multitasking cost) between the pre- and post-training sessions and the pre- and ~1-mo follow-up session. This method was used as we had specific hypotheses for the double-blind groups and active controls. For all transfer tasks, key performance metrics were compared between the pre- and post-training sessions as well as the pre- and 1-mo follow-up sessions to evaluate the presence of training transfer.

### Multitask training

The key training task involved a sensory-motor response selection task (Filmer, Lyons, et al. 2017; Ehrhardt et al. 2021), consisting of either a centrally presented colored circle (red: RGB 237 32 36, dark green: RGB 10130 65, or dark blue: RGB 44 71151, subtending approximately 2.7° of visual angle) and/or 1 of 3 complex tones (as used in Dux et al. 2006). Participants responded by pressing designated keys on a keyboard, with each stimulus mapped to a specific key (A, S, D [left hand] J, K, L [right hand]; with the index fingers on D and J keys, respectively). The task included 3 different trial types: single-visual (a colored circle; red, green, or blue), single-auditory (1 of 3 complex tones), or a combined visual–auditory multitask (1 colored circle and 1 complex tone). Multitask trials had both stimuli presented simultaneously (0 ms stimulus onset asynchrony), consistent with previous multitasking research (Dux et al. 2009). For both single and multitask trials participants were instructed to respond as quickly and as accurately as possible to the presented stimuli. On dual-task trials, they were instructed to respond to both stimuli at the same time, resulting in 2 separate key presses. RTs for key presses were recorded and used for subsequent analyses. Trials proceeded with a fixation square (0.4° diameter) centrally presented for either 600 or 1,000 ms (randomly jittered), followed by a 200 ms stimulus presentation. Visual stimuli appeared centrally, while auditory stimuli played in stereo through Sure SRH440 over-ear headphones. Participants had 2,200 ms from stimulus onset to respond. Response mappings were counterbalanced across participants and groups, with half using their left hand for the auditory task and right hand for the visual task, and vice versa for the other half. In the pre-training session, participants practiced both single- and multitasks until they achieved a 70% accuracy cutoff for the multitask condition, minimizing exclusion rates due to poor performance. The initial practice comprised 3 blocks: the first 2 blocks containing 15 trials of each single task (auditory, then visual), and the third block featuring 30 trials with all trial types (single visual, single auditory, and multitask) presented 10 times each, in random order. If participants failed to reach 70% accuracy, they repeated the third block until the threshold was met. In all sessions (pre-, post-, follow-up, and training sessions), participants completed 240 trials, equally divided among the 3 trial types (80 single-visual, 80 single-auditory, and 80 multitask) with a 30-s break at the halfway point. Before each subsequent session, participants completed block 3 of the practice session to refresh response mappings (10 trials each: single-visual, single-auditory, and multitask). The training aimed to increase the speed with which participants could execute single or multiple decisions (indicating more efficient information processing). Each 240-trial session lasted ~13 min. The control training task group, who trained on a RSVP task (described below), also performed multitasking paradigm during pre-, post-, and follow-up testing sessions.

### RSVP training task (control)

To evaluate whether any potential advantages of combining stimulation and training are specifically related to multitasking training, an active control group trained on a selective attention control task: a modified RSVP task (Potter and Levy 1969; Raymond et al. 1992; Dux and Marois 2009). Participants were shown a fixation square of 0.4° diameter for a randomly determined duration of either 0.2, 0.3, 0.4, 0.5, or 0.6 s. This was followed by a rapid stream of 7 distractor numbers, with the aim being to identify a single target letter embedded within the stream (e.g. 4, 2, W, 5, 3, 8, 9, 7). The height of the numerical and alphabetical stimuli was ~0.8° of visual angle. Responses were made via keypress on a standard keyboard corresponding to the displayed letter (possible letters presented: “A,” “B,” “C,” “D,” “E,” “F,” “G,” “H,” “J,” “K,” “M,” “N,” “P,” “R,” “S,” “T,” “W,” “Y,” “Z”). Letter position was randomly allocated throughout the session between positions 3 and 6 in the serial presentation. The task consisted of 240 trials, divided into 3 blocks. Each rest period between blocks was dynamically adjusted to ensure task duration (time spent training) matched the single/multitask that was trained. The presentation duration was dynamically adjusted for each participant to maintain accuracy at ~70%. In the pre-training session, each stimulus was initially presented with a 100 ms duration, then reduced in 10 ms increments if participants accurately responded to 5 consecutive trials. If 2 incorrect trials occurred successively, the presentation duration would increase by 10 ms per stimulus. The minimum stimulus duration was 10 ms. Each subsequent session began with the final presentation duration (i.e. 20 ms) of the pre-training session. Presentation duration served as the dependent variable, with shorter durations indicating better performance. The key distinction between this task and the single/multi response selection training task is that accuracy and presentation duration were the main performance measures, rather than response time. Thus, this task did not require or test for the speed of responses. Furthermore, this task has forward and backward masking of the target stimulus that limit performance. Each 240-trial session lasted ~13 min. This task was also used in the pre-, post-, and follow-up testing sessions as a transfer task for the 4 groups that trained on the multitasking paradigm.

### Visual search

We employed a visual search task (locating a “T” among distractor “L”s) as we have observed transfer to this domain in 2 previous experiments that combined tDCS with cognitive training (Filmer, Lyons, et al. 2017; Filmer, Varghese, et al. 2017). Trials commenced with a fixation dot of 0.25° diameter, lasting for a randomly determined duration of either 0.4 or 0.6 s. Subsequently, the fixation cross vanished, and an array of “L” stimuli plus a single target “T” stimulus appeared (total array subtended 17° of visual angle). These stimuli were displayed equidistant from one another in random positions within the search array. Participants needed to find the “T” rotated either 90° or 270°, among randomly rotated—either 90° or 270°—distractor letter ‘L’s. The 2 stimuli had an approximate line width subtending 0.2° and a height of 0.8° of visual angle. Task difficulty was manipulated by varying the number of distractor “L”s: either 7, 11, or 15, with 80 trials for each set size, totaling 240 trials. Participants had 3,000 ms to press the “Z” key with their left index finger if the “T” was rotated by 270° or the “M” key with their right index finger if the “T” was rotated 90°. Practice consisted of 15 trials before each of the pre-, post-, and follow-up testing sessions; feedback was provided for incorrect responses during practice in the form of a brief auditory tone.

### Functional MRI task

The task performed within the scanner was similar to the response-selection training task paradigm described above. To optimize the task for the MRI environment and enhance blood oxygen level dependent (BOLD) signal detection, we implemented minor modifications. Specifically, participants performed solely the single-task component, presented in blocks of 4 trials. Dual-task trials were not included because individual BOLD responses for each task cannot be resolved in dual-task trials. Within each block, stimuli were randomly presented but were of the same modality. A 12-s rest period separated each block. Participants completed a total of 8 blocks per modality in each run, with 3 runs in total for each session, resulting in 96 trials per modality per session.

### Imaging acquisition

Scans were acquired with a MAGNETOM 7T Plus MRI scanner (Siemens Healthcare, Erlangen, Germany), equipped with a 64-channel receive head coil (Nova Medical, Wilmington, MA, USA). Anatomical T1w images were acquired with a magnetization-prepared 2 rapid acquisition gradient echo sequence for ultra-high-resolution structural images: 0.75 mm^2^ isotropic, TR = 4,300 ms, TE = 3.38 ms. High temporal and spatial resolution functional images were acquired using a 1.8 mm^2^ isotropic voxel echo planar imaging multiband fMRI sequence; TR = 1,000 ms, TE = 19.4 ms, field of view = 192 x 192 mm, flip angle = 60°, 57 interleaved slices (1.8 mm thick), providing whole-brain coverage. Stimulus presentation was synchronized with volume acquisition. Participants lay supine in the scanner and viewed the visual display via rear projection onto a mirror mounted on the head coil. Other scans not reported on in the current study were also acquired.

### Imaging data

Results included in this manuscript come from preprocessing performed using fMRIPrep 20.2.3 (Esteban et al. 2019), which is based on Nipype 1.6.1 (Gorgolewski et al. 2011). The preprocessing details below were modified from the fMRIPrep html output boilerplate for a typical participant. The full output is pasted verbatim in the Supplementary material as recommended for reproducibility (Esteban et al. 2019). This boilerplate was generated for 1 representative participant, so the total number of T1w images and functional runs differed whenever a participant with missing scan/s was processed.

### Structural MRI preprocessing

For full details of anatomical MRI preprocessing, see the Supplementary material. In short, T1w images were corrected for intensity non-uniformity, skull-stripped, segmented into cerebrospinal fluid, white-matter and gray-matter, and normalized to Montreal Neurological Institute (MNI) space. This was all performed within the fMRIPrep pipeline which included the following functional data preprocessing.

### Functional MRI preprocessing

For full details of functional MRI preprocessing, see the Supplementary material. In short, each of the 6 BOLD runs per session were preprocessed separately for each participant. First, a reference volume and its skull-stripped version were generated. Then a deformation field to correct for susceptibility distortions was estimated, and the BOLD reference scan was co-registered to the T1w reference. Head motion parameters were then estimated, before any spatiotemporal filtering. Slice timing correction was then applied, and the corrected BOLD time-series was resampled onto their original, native space.

### Univariate fMRI analysis

Custom MATLAB (The MathWorks Inc., 2019) batch scripts were used to conduct all univariate analyses, using statistical parametric map (SPM) 12 functions and the Marsbar toolbox (Brett et al. 2002). The functional MRI data (only for the univariate analysis) were first smoothed using a 6 mm Gaussian kernel to reduce spatial noise. We then carried out a first-level within-subject analysis where BOLD signal change was modeled for each single-task performed in the scanner.

As visual or auditory tasks were executed within blocks of 4 unimodal trials, these blocks were modeled through a general linear model as box-car functions, convolved with a canonical hemodynamic response function and its temporal and dispersion derivatives. We also included regressors for 6 motion parameters (X, Y, Z, pitch, yaw, roll). Each block was modeled as a single trial of 12 s duration (4 trials of 3 s each). This approach was designed to improve the signal-to-noise ratio for each block.

We then conducted a group level SPM analysis on the pre-training and post-training fMRI data separately to identify the regions of interest (ROIs) for subsequent analyses. The coordinates for the maximally activated voxel within a cluster of voxels (10 voxel cluster extent threshold, uncorrected *P*-value = 0.001) that exhibited increased activity for both auditory and visual stimulus blocks, relative to rest periods, were taken to define the ROIs. These ROIs were defined based on the pre-training conjunction map, but using the post-training conjunction map did not result in different ROIs.

### Multivariate pattern analysis

To conduct the subsequent multivariate pattern analysis (MVPA) using a linear support vector machine (SVM) on these ROIs separately, as in Garner and Dux (2015), we defined 7 and 9 mm radius spheres around the voxel coordinates of the activity peak in each cluster from the conjunction analysis, and a mask was created for each of these sizes for each ROI using the Marsbar toolbox (Brett et al. 2002). Several of the 9 mm ROIs overlapped in both cortical (e.g. parietal lobe) and subcortical (e.g. putamen) regions; therefore, we focused the analysis on the 7 mm ROIs. We observed only a partial convergence in the results across these two ROI sizes for a subset of the ROIs.

MVPA was implemented using the Pattern Recognition for Neuroimaging Toolbox (PRoNTo version 3, Schrouff et al. 2013), in MATLAB (The MathWorks Inc., 2019). Prior to each multivariate pattern analysis, the data for each voxel in a ROI were z-transformed and mean-centered. This involved subtracting the condition mean for the entire ROI from the response in each individual voxel, which controlled for overall differences in signal amplitude between conditions. We trained a series of binary classifiers to differentiate between patterns of activity linked with the visual and auditory single tasks performed in the scanner. Using a leave-one-run-out cross-validation method, for each iteration 1 run was held out to evaluate the classifiers’ generalization performance, and the remaining 2 runs were used to train the classifier. Decoding accuracy was averaged across each of these cross-validation loops, for each ROI, at each session. Post- minus pre-training decoding accuracy was calculated to obtain the change in decoding for each ROI for each participant after training. To ensure the suitability of the data for the subsequent correlational analyses, outliers in the pre-training data for each ROI were removed. Specifically, if a participant’s data for a particular ROI exceeded 3 standard deviations from the whole sample’s mean for that ROI, it was removed. This resulted in 65 data points being removed (out of 12,880—amounting to ~0.5% of the data).

### Functional MRI motion analysis

Along with clear instructions to participants to minimize movement as much as possible, we applied a head motion feedback technique to reduce head movement in the scanner (Krause et al. 2019). This involved placing medical tape across participants’ forehead, connected to the head coil, with the goal of providing tactile feedback from movement rather than meaningfully constricting movement. We also employed stringent motion correction and exclusion measures to minimize the influence of motion-related artifacts. Volumes with a frame-wise displacement exceeding 0.2 mm were removed as outliers due to excessive motion (refer to Power et al. 2014). Runs containing 50% or more motion outlier volumes were excluded (Murphy et al. 2020). If a participant had two or more runs excluded from a session, their data from that session were removed from subsequent analyses pre- vs post-training. As a result, the data from 11 participants were excluded from analyses for at least 1 session.

### Functional MRI behavior analysis

Changes in decoding in each of the defined ROIs were separately correlated (using Bayesian correlations, custom python script) with the changes in performance on the relevant behavioral metrics. Using the same criterion as for the behavioral analyses, a BF_10_ > 3.2 was taken to indicate meaningful evidence for the alternative hypothesis—in this case that there was a correlation between the change in decoding in an ROI and the change in performance on a particular behavioral dependent variable—while a BF_10_ < 0.3125 signified evidence for the null hypothesis (no correlation). To assess whether these correlations differed between stimulation groups, we used a Fisher-Z transformation of the correlation coefficients and performed a *t*-test on these values. The resulting *P*-values were false discovery rate (FDR) corrected and any remaining comparisons with a *P*-value < 0.05 were interpreted as indicating significantly different correlations.

## Results

### Overview

The data used here are the same as used in Wards et al. (2023). We trained 178 participants (aged 18 to 40; *M* = 22.86, SD = 3.93 yr, 119 females) on a multitasking protocol shown to result in transfer of performance gains to visual search when combined with tDCS (Filmer, Lyons, et al. 2017). Our pre-registered study (https://tinyurl.com/5h8u72j5) spanned 10 sessions: an initial pre-training behavioral assessment, 2 MRI sessions pre-training, 4 days of tDCS-assisted training, a post-training MRI session, post-training behavioral analysis, and concluding with a follow-up behavioral assessment 30 days later (Fig. 1).

The current study assesses brain activity recorded pre- and post-training (sessions 3 and 8), and changes in behavior from the pre-training behavioral session to the post-training behavioral session. RT and accuracy differences across time were measured via a range of behavioral tasks assessing working memory, attention, response inhibition, and multitasking. Full details of all tasks included in the battery can be found in Ehrhardt et al. (2024). The present investigation focuses primarily on the multitasking training task, and a visual search transfer task. Briefly, the multitasking paradigm required participants to respond to either an auditory or visual stimulus on single-task trials, or both an auditory and visual stimulus on dual-task trials. The visual search task required searching for a rotated letter “T” among a varying number of distractor letter “L”s. We focused our analyses on the fMRI component of the imaging data, applying MVPA to the BOLD signal during single-task performance of the multitasking paradigm. Decoding accuracy served as a proxy for the extent of overlap of task-relevant activity, which we examined pre- and post-training. In addition, we adopted an individual differences approach which allowed us to capitalize on the interindividual variability observed in the neurophysiology underlying cognitive task performance (Crittenden and Duncan 2014; Garner and Dux 2015), as well as training and tDCS outcomes (Kanai and Rees 2011; Mayhew and Kourtzi 2013; Jaeggi et al. 2014; Sampaio-Baptista and Johansen-Berg 2017).

Participants were divided into 5 groups to dissect effects of stimulation region, intensity, and task specificity (Fig. 1). The groups are as follows:

1. Sham group: underwent sham stimulation with multitasking training, serving as the tDCS control.
2. 1 mA LH group: received 1 mA stimulation to the left PFC with multitasking training.
3. 1 mA RH group: 1 mA stimulation to the right PFC with multitasking training, for comparison to the 1 mA LH group to assess stimulation location specificity.
4. RSVP group: 1 mA stimulation to the left PFC but trained on a RSVP paradigm, for comparison to the 1 mA LH group to determine training task specificity.
5. 2 mA LH group: 2 mA stimulation to the left PFC with multitasking training, to be compared to the 1 mA LH group for examining effects of different stimulation intensities.

Comparisons were also made between 1 mA LH, 1 mA RH, and 2 mA LH against the sham group for a complete understanding of stimulation effects on both trained and untrained tasks. Bayesian *t*-tests were used for all planned comparisons, with BF values indicating the strength of the evidence for the alternate hypothesis (BF_10_ > 3.2; Kass and Raftery 1995). Deviating from our preregistration, only Bayesian results are reported (instead of both Bayesian and null hypothesis significance testing [NHST] tests), as they are fundamentally more conservative and so reduce the likelihood of type 1 errors (Gelman and Tuerlinckx 2000). Nevertheless, the patterns of results using both Bayesian and NHST methods were consistent.

### Combined tDCS and multitasking training led to transfer

Full details of analyses and results beyond the scope of the present investigation can be found in Wards et al. (2023). Here we recap the key relevant behavioral findings. We first ensured comparable baseline performances across the participant groups via a pseudo-randomization procedure for participant allocation. Our analyses revealed strong evidence against pre-training differences among the groups for the relevant trained and untrained task metrics (BF_10_ < 0.085 for all comparisons—see Wards et al. 2023).

**Fig. 2:**
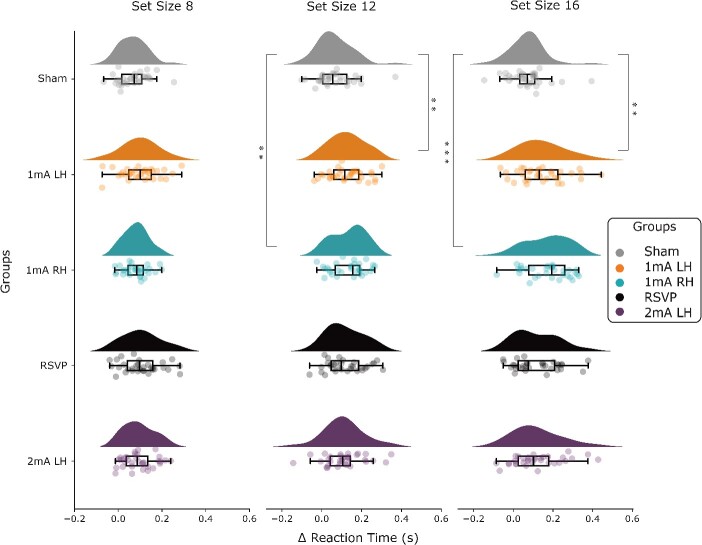
Prefrontal tDCS results in enhanced visual search performance*.* Changes in visual search by group and set size (as previously reported in Wards et al. [2023]). All 5 groups show similar changes in RTs for set size 8 trials. However, both 1 mA left hemisphere (1 mA LH) and right hemisphere (1 mA RH) tDCS groups that trained on multitasking have greater performance improvements than sham for set sizes 12 and 16. Each point represents an individual’s change in RT from pre–post training. Positive values reflect an improvement in performance from pre–post training. Δ = change in, ** = strong evidence, *** = decisive evidence (Kass and Raferty 1995) for a difference between groups.

Turning to training effects, multitasking training enhanced performance as indicated by a larger reduction in RTs pre- to post-training for both single- and multitask trials for the 1 mA LH group compared to the RSVP training group (BF_10_ = 38.182, error < 0.001%; BF_10_ = 313.222, error < 0.001%). There were only faint differences in changes in multitasking cost (the difference between RTs on single stimuli trials compared to RTs on the dual stimuli trials) between 1 mA LH multitasking training vs the RSVP group (BF_10_ = 2.640, error < 0.001%). Notably, despite observing an impact of tDCS on transfer performance (see below) neither tDCS site nor dose differentially impacted multitasking performance improvements. Specifically, comparing the 1 mA LH, 1 mA RH, and 2 mA LH groups to sham showed no effect of stimulation on performance (single-task RTs: all BF_10_ = 0.248 – 0.275, errors < 0.009%; multitask RTs: all BF_10_ = 0.247 – 0.620, errors < 0.01%; multitasking cost: all BF_10_ = 0.293 – 1.190, errors < 0.009%; see Wards et al. 2023). Crucially, we did observe transfer effects. To investigate the influence of paired training and stimulation on transfer effects, performance on each task for the 1 mA LH, 1 mA RH, and 2 mA LH groups were compared to sham across the entire behavioral battery. We found that participants receiving 1 mA stimulation to the left or right PFC, in conjunction with multitasking training, showed enhanced performance in the visual search task, compared to sham (Fig. 2). Specifically, these gains were observed for RTs on trials with set sizes 12 and 16 [BF_10_ = 11.410, BF_10_ = 13.215 (1 mA LH vs sham); BF_10_ = 14.915, BF_10_ = 122.788 (1 mA RH vs sham), errors all <0.003%]. Furthermore, these effects endured for 1-mo post-training for set-size 16 for both the left and right 1 mA PFC stimulation groups relative to sham (BF_10_ = 6.080 and 54.284, respectively, errors < 0.004%; see Wards et al. 2023). There was inconclusive to moderate evidence against an effect of stimulation and training on all other tasks in the battery (BF_10_ = 0.244 – 1.250), highlighting the specificity of the transfer to visual search observed here.

### Changes in decoding accuracy underly transfer

Our primary objective was to ascertain whether changes in decoding accuracy within multitasking task-relevant regions could predict changes in performance on the transfer task. Prior research has found that improvements in multitasking via training are associated with a separation of the neural activity patterns corresponding to single-task performance, in a frontal–parietal–subcortical subset of brain regions (Garner and Dux 2015). As activity within such areas scales with the complexity of many cognitive tasks (Chein and Schneider 2005; Duncan 2010; Fedorenko et al. 2013; Crittenden et al. 2016), including visual search (Jung et al. 2021), and that Filmer, Varghese, et al. (2017) found that left PFC anodal stimulation resulted in faster evidence accumulation for both a response selection task and visual search task, we hypothesized that paired training and tDCS specific alterations in the representation of the training task could underpin generalized performance improvements to visual search.

To isolate task-relevant regions, we tested for brain regions that showed increased activity for both single-tasks from the multitasking training paradigm, relative to fixation (conjunction contrast). This resulted in identification of 41 regions across frontal, parietal, cerebellar, and basal ganglia locations (see Supplementary Table S1). These regions broadly align with those identified in earlier studies investigating executive operations (Chein and Schneider 2005; Dosenbach et al. 2007; Duncan 2010; Fedorenko et al. 2013; Crittenden et al. 2016).

Having identified ROIs that respond to both tasks, we next sought to identify which of those regions showed training-related changes in task representation that corresponded to performance changes on the visual search task. To achieve this, we trained a linear SVM algorithm to classify between single-task activity patterns, using voxels in each ROI as features. We then calculated the change in decoding accuracy from pre- to post-training for each ROI and correlated these changes with the performance changes on the visual search task within each group. These correlations were compared to sham using the Fisher-Z transformation method, and *P*-values were corrected for multiple corrections using the FDR method.

**Fig. 3:**
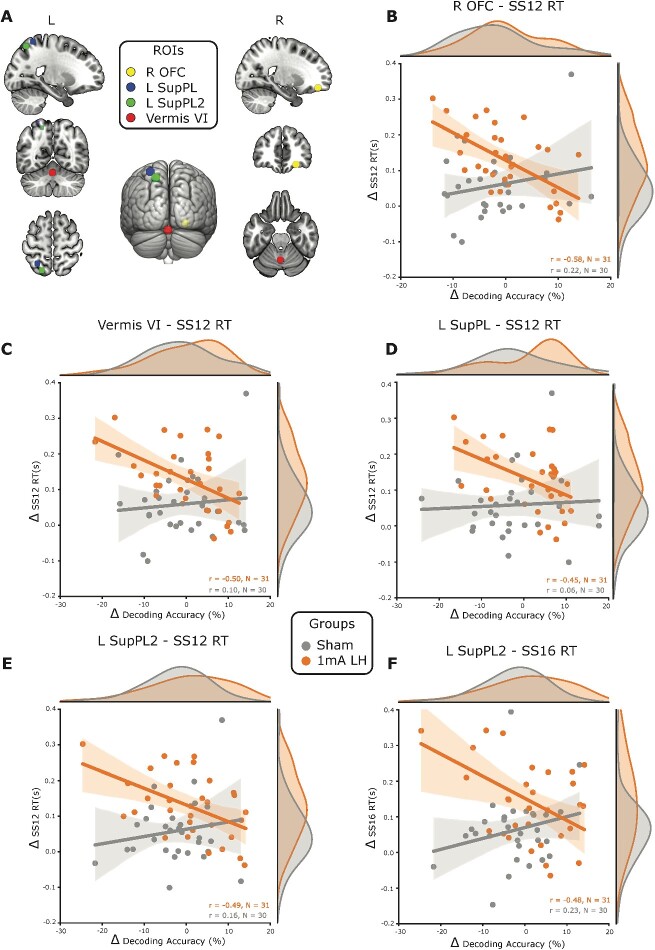
Decreased decoding accuracy for the trained task is associated with improvements on untrained visual search performance*.* A) ROIs that were conjointly activated by the trained task subcomponents, and that showed significantly greater correlation coefficients between changes in decoding accuracy and visual search performance changes than sham. These regions were the right orbitofrontal cortex (R OFC), cerebellum vermis VI (vermis VI), and 2 regions in the left superior parietal lobe (L SupPL and L SupPL2). B) For the group that received left PFC anodal tDCS, the R OFC changes in decoding accuracy were negatively correlated with the changes in RT on visual search trials with 12 distractors (SS12) and this correlation was significantly different to sham. This same pattern of results with negative correlations between changes in decoding accuracy and changes in set size 12 RTs was observed for the vermis C) VI, D) L SupPL, and E) L SupPL2. The L SupPL2 region also had a negative correlation between changes in decoding accuracy and changes in set size 16 RTs. F) Shading around the regression lines displays 95% confidence intervals. SS12 = set size 12, SS16 = set size 16, L = left, R = right, *r* = Pearson’s correlation coefficient, *n* = sample size, Δ = change in.

There was moderate to strong evidence, in a subset of the identified regions (Fig. 3A), which showed that increases in decoding accuracy pre- to post-training were negatively correlated with improvements on the visual search performance (Fig. 3B–F) for individuals that received 1 mA left prefrontal stimulation. Specifically, enhancements to visual search performance corresponded to tDCS and training induced decreases in the differentiability of single-task activity in these regions. The anterior cerebellum vermis VI lobe (*r* = −0.50, BF_10_ = 11.1), two separate regions within the left superior parietal lobe (*r* = −0.45, BF_10_ = 5; *r* = −0.49, BF_10_ = 10.2), the right inferior parietal lobe (*r* = −0.45, BF_10_ = 5.2), and the right orbitofrontal cortex (r = −0.58, BF_10_ = 64.7) all showed negative correlations between changes in decoding accuracy and changes in visual search RTs for set size 12. While multiple regions demonstrated negative correlations between changes in decoding accuracy and RTs for set size 12, only 1 of the 2 left hemisphere superior parietal lobe regions also showed evidence of a negative correlation between decoding accuracy changes and set size 16 RTs (*r* = −0.48, BF_10_ = 7.6). Except for the right inferior parietal lobe, all the correlations in the regions presented here were different from sham (FDR corrected *P* < 0.05 for all comparisons). Notably, no correlations between changes in decoding accuracy and changes in visual search performance were statistically different compared with sham for the right hemisphere 1 mA prefrontal stimulation group, the 2 mA left prefrontal stimulation group, or the RSVP group.

### Changes in decoding accuracy were not predictive of multitasking cost improvements

Following the findings from Garner and Dux (2015), we combined all 4 groups that trained on the multitasking task and compared them to the group that trained on the RSVP task. As the 2 groups differed significantly in sample size [multitasking group (MT); *n* = 122, RSVP; *n* = 32], we used a Levene’s test to check the equality of the variance within each ROI between the two groups (*P* > 0.05 for each comparison). Next, we averaged the change in decoding accuracy across the FP–SC regions in our sample that were the closest (in MNI coordinates) to those identified in Garner and Dux. Closeness was assessed by proximity in mm. This was done by firstly using the BioImage Suite Web app to convert Talaraich coordinates from Garner and Dux (2015) into MNI coordinates. Where multiple ROIs from either Garner and Dux or the current study were labeled as the same region, the MNI coordinates for these regions were compared and the ROIs that were the closest in mm in the *X*, *Y*, and *Z* planes were selected. We then calculated the correlation between changes in multitasking cost and changes in decoding in this FP–SC network within the combined MT, and the RSVP training group. In contrast to Garner and Dux, we found evidence for there being no correlation between changes in decoding in the FP–SC network and changes in multitasking cost for both groups (MT, *r* = 0.121, BF_10_ = 0.271; RSVP, *r* = −0.045, BF_10_ = 0.222). This lack of evidence for a change in representational content related to the multitasking component tasks within this FP–SC system in these groups is perhaps not surprising given we did not observe meaningful improvements in multitasking cost for any group that received tDCS. This likely reflects that only approximately one-third of the number of training trials used in Garner and Dux were used here, although there were other design differences, so definitive conclusions cannot be made on this.

## Discussion

We examined how combining prefrontal tDCS and multitasking training changes the neural representations of the trained tasks to give rise to generalizable learning. We found that pre- to post-training decreases in the discernibility between trained task representations were associated with faster visual search performance for the group that received left prefrontal tDCS. This association was specific to the left superior parietal lobe, right orbitofrontal cortex, and the cerebellum vermis. By characterizing changes in multivariate patterns of activity that occur across task exposure, we infer that task representations are modified by the specific combination of PFC tDCS and multitasking training, resulting in greater performance transfer. Therefore, the present results inform models regarding the neural substrates of computations that underly multitasking and skill generalization. Musslick et al. (2017) demonstrated, via neural network modeling, that shared task representations improve learning generalizability to the detriment of multitasking ability. The current findings support this framework, and provide an intriguing suggestion that the addition of tDCS during learning, in healthy humans, may induce greater sharing of task information, thereby enhancing learning generalization.

Using a preregistered study design, large sample size (*n* = 178), double blinding of stimulation condition, and active control groups, we have made it possible to rule out common issues that often limit conclusions from tDCS studies (Polanía et al. 2018; Filmer et al. 2019). Specifically, we double blinded the 1 mA LH and sham groups; thus, differences in performance between these 2 groups cannot be attributed to experimenter bias or participants’ expectations of effects. Our active training task control group—RSVP—that also received 1 mA LH stimulation did not show training transfer. Therefore, it was the specific combination of multitasking training with tDCS that resulted in improved visual search speed. The lack of transfer for the 2 mA LH group excludes the role of peripheral (Vanneste et al. 2020) or arousal (McIntire et al. 2014) effects of stimulation. With regards to the MVPA data quality, we used an in-scanner motion reduction technique and conservative motion exclusion thresholds, reducing the likelihood of spurious associations due to imaging artifacts. The current findings therefore reinforce the potential effectiveness of tDCS, while also highlighting the dependency of induced effects on the concurrent task being performed, and the intensity of stimulation (Hoy et al. 2013; Martin et al. 2014; Ehrhardt et al. 2021). Finally, our neuromodulation interventional study design, using established computerized cognitive measures, enhances the reliability of our findings, and avoids issues related to sample size pervasive in other types of brain-behavior studies (Marek et al. 2022).

Previously, the reduction of multitasking costs via extensive training was found to be associated with greater segregation of task-related information processing in predominantly frontal, parietal, and subcortical regions (Garner and Dux 2015). However, we found no reduction in multitasking cost after training for any of the groups that received tDCS. It may simply be that the training protocol used in the current study—which had one-third of the trial numbers used in Garner and Dux (2015)—was not sufficient to induce the appropriate learning for such associations to be observed. However, purely training induced cost improvements were observed for the sham group here (Wards et al. 2023). In the most similar experiment to the present study, transfer induced by tDCS was also found without the stimulation concurrently benefitting the trained task (Filmer, Lyons, et al. 2017). In light of recent findings regarding the computational and neurobiological substrates of information sharing (Musslick et al. 2017; Vaidya et al. 2021), and the theoretical framework proposed in Garner and Dux (2023), as well as in Musslick and Cohen (2021), these findings are not entirely surprising. Indeed, this prior work points toward multitasking ability being inversely associated with the ability to generalize related skills. Convergent with this literature, Mill and Cole (2023) show that representations shift from context independent and task-general, to task-specific during learning. Interpreting our findings with regards to these learning related representational dynamics; tDCS, through its effects on learning processes (Simonsmeier et al. 2018), may be stalling these dynamics, holding them in an “early learning” state where activity patterns are less specific to the task at hand and more generalizable to another task. Future work is required to discern the potential different time scales for task-specific and transfer learning effects in the brain and the potential disruptive effects of tDCS on task-specific training.

The influence of paired training and tDCS on the visual search task were present for both the left and right hemisphere 1 mA groups; however, we only found evidence that left hemisphere tDCS induced changes to task representations that were associated with the visual search improvements. This may reflect dissociable mechanisms for transfer based on the prefrontal circuitry that is stimulated. The left and right prefrontal cortices have been differentially implicated in many cognitive operations, such as motor sequence learning (Greeley and Seidler 2019), multitasking (Dux et al. 2006; Garner et al. 2020), planning (Kaller et al. 2011), and relational integration (Bunge et al. 2008). Indeed, the integration of relationships between stimuli, or in other words, the abstract rules associated with the task, has been found to be encoded in the left PFC (Badre and D’Esposito 2007; Badre et al. 2010). Meanwhile, the sharing of these abstract rules has been proposed as a mechanism for transfer (Garner et al. 2016; Vaidya et al. 2021; Garner and Dux 2023). It may be that the application of tDCS to this region enhances its capacity for the sharing and integration of information pertaining to task contingencies, within task-general frontal, parietal, and cerebellar regions, resulting in enhanced generalization of learning. However, none of the other 7 tasks tested demonstrated an effect of tDCS on generalizable improvements in RTs. It may be that the specific cognitive processes taxed during the visual search task rendered it the only task sensitive enough to detect an effect; greater tDCS specific improvements on the most difficult condition in this task supports this account.

It is also conceivable that tDCS facilitated learning generalization via improvements on a shared cognitive component of the trained task and visual search. For example, the dorsolateral PFC—used as the focal point of stimulation in the current study—is involved in evidence accumulation processes (Philiastides et al. 2011) and the speeding up of information processing after training (Dux et al. 2009). Using a linear ballistic accumulator to model response times, Filmer, Varghese, et al. (2017) found that dorsolateral PFC tDCS induced improvements on both the trained task and visual search task were related to improvements in information processing efficiency in both tasks. In combination with the current results, these findings suggest that the trained-on multitasking paradigm and the visual search task share central information processing resources, perhaps related to stimulus–response mapping (Jiang and Kanwisher 2003), that can be reduced with combined tDCS and training. Future work is needed to disentangle the specific action of tDCS on the cognitive components associated with performing these tasks.

Considering the broader context of non-invasive brain stimulation, transcranial random noise stimulation (tRNS) has also shown promise in enhancing learning generalization. The stochastic nature of tRNS is believed to promote a more generalized state of neural activity, avoiding an overfitting to specific stimuli (Battelli et al. 2022; Cappelletti et al. 2013) resulting in performance transfer. The application of tDCS might share a similar mechanism, with the current results supporting the account of stimulation reducing an overfitting of neural activity. However, there are important differences between tDCS and tRNS. Specifically, tDCS can be thought of as providing a specific subthreshold shift in the membrane potential of neurons in the regions affected by the exogenous electric field. By introducing a consistent subthreshold modification of the membrane potential, this allows for a potentiation of ongoing neural activity, in both spike frequency and timing (Radman et al. 2007; Reato et al. 2010; Bikson and Rahman 2013). This contrasts with tRNS, which due to the random fluctuations in the electric field, may not provide a consistent enough shift in the membrane potential to increase the likelihood of an action potential specifically in neurons within the active neural circuitry, as tDCS does (although, see Fertonani et al. 2011). While subthreshold shifts in membrane potential may underlie the effects of both techniques, many studies have demonstrated contrasting effects of tDCS and tRNS on cortical excitability (see Reed and Cohen Kadosh 2018 for a review). The differences in the consistency of the electric fields induced by the 2 techniques, and how these differentially interact with endogenous neural activity, may be the crucial factor differentiating their mechanisms. Both forms of stimulation may prove useful in boosting transfer, with their subtly different mechanisms possibly lending an advantage in different applications. Further research is required to narrow in on the contexts in which different stimulation types may be able to enhance learning generalization.

In conclusion, our findings underscore that coupling PFC tDCS with multitasking training modifies the dynamics of task representations, which corresponds to learning generalization to visual search. These results resonate with modern theories highlighting the fundamental role of shared task-representations in knowledge generalization.

## Supplementary Material

CerCor-2024-00044_SupplementaryMaterials_bhae209
